# Activation of perineuronal net-expressing excitatory neurons during associative memory encoding and retrieval

**DOI:** 10.1038/srep46024

**Published:** 2017-04-05

**Authors:** Shota Morikawa, Yuji Ikegaya, Minoru Narita, Hideki Tamura

**Affiliations:** 1 Laboratory of Gene Regulation Research, Graduate School of Biological Sciences, Nara Institute of Science and Technology (NAIST), 8916-5, Takayama, Ikoma, Nara 630-0192, Japan; 2 Life Science Tokyo Advanced Research Center (L-StaR), Hoshi University School of Pharmacy and Pharmaceutical Sciences, 2-4-41, Ebara, Shinagawa-ku, Tokyo 142-8501, Japan; 3 Laboratory of Chemical Pharmacology, Graduate School of Pharmaceutical Sciences, University of Tokyo, 7-3-1, Hongo, Bunkyo-ku, Tokyo 113-0033, Japan; 4 Center for Information and Neural Networks, National Institute of Information and Communications Technology, Suita City, Osaka, 565-0871, Japan; 5 Department of Pharmacology, Hoshi University School of Pharmacy and Pharmaceutical Sciences, 2-4-41, Ebara, Shinagawa-ku, Tokyo 142-8501, Japan

## Abstract

Perineuronal nets (PNNs), proteoglycan-rich extracellular matrix structures, are thought to be expressed around inhibitory neurons and contribute to critical periods of brain function and synaptic plasticity. However, in some specific brain regions such as the amygdala, PNNs were predominantly expressed around excitatory neurons. These neurons were recruited during auditory fear conditioning and memory retrieval. Indeed, the activation of PNN-expressing excitatory neurons predicted cognitive performance.

Some neurons are enwrapped by a specialized matrix structure called a perineuronal net (PNN), which acts as a diffusion barrier for ion channels and receptors, scaffold binding for signaling molecules, and an ionic buffer^1^. Therefore, PNNs modulate synaptic transmission and neuronal activity^2^. PNNs in the adult brain are expressed in a region- and cell-specific manner; in particular, they are expressed around parvalbumin (PV)-positive inhibitory neurons^3^,^4^, although some glutamatergic neurons express PNNs^5^. Recent studies using enzymes that degrade PNNs demonstrate that PNNs regulate synaptic plasticity, critical period closure, and fear memory^6^,^7^,^8^. Despite these important functions, it is unclear what differentiates neurons with PNNs from those without. Here, we show that PNNs are mainly expressed around excitatory neurons in brain regions related to emotional learning and memory. We then compared the expression of c-Fos, a marker of neuronal activity^9^, between neurons with and without PNNs after fear conditioning, which is a form of associative learning^10^.

## Results

### PNNs are predominantly expressed around excitatory neurons in specific brain regions, including the amygdala

**Figure 1:**
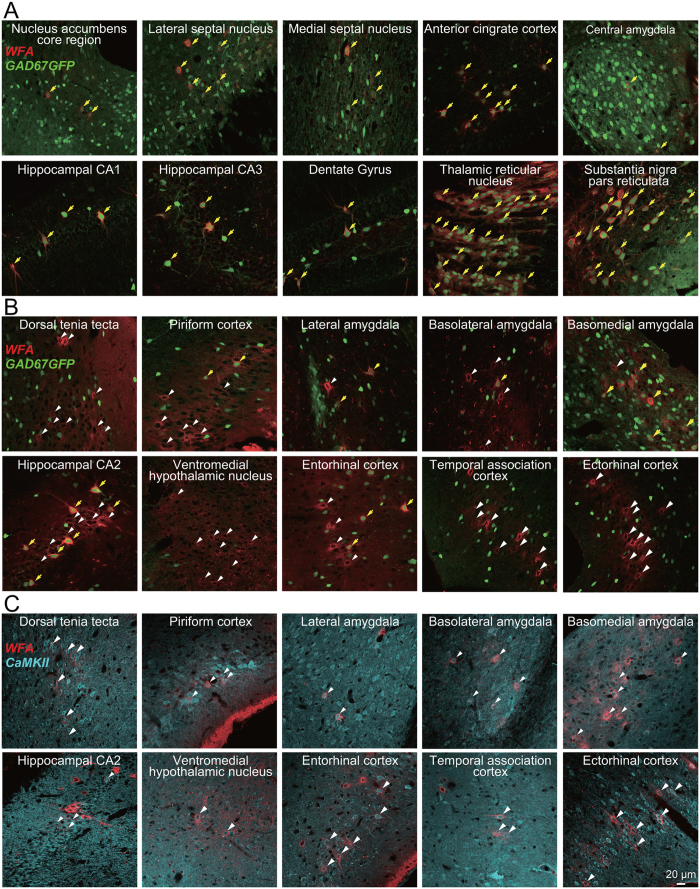
Distribution of PNNs throughout the whole mouse brain. Merged images showing signals generated by WFA (red), GFP (green), or an anti-CaMKII antibody (cyan) in GAD67-GFP knock-in mice (**A**,**B**) or C57BL/6J mice (**C**). A subpopulation of GFP-expressing GABAergic neurons is enwrapped by WFA-positive PNNs (yellow arrows; **A**,**B**); however, WFA-positive PNNs also enwrap some neurons that do not express GFP (white arrowheads; **B**) or neurons that express CaMKII (white arrowheads; **C**).

We investigated the overall distribution of PNN using *Wisteria floribunda* agglutinin (WFA), a ubiquitous marker for PNNs^11^, in GAD67-GFP knock-in adult mice in which GABAergic neurons are specifically labeled by GFP^12^. These mice have a normal GABA content in the adult brain and exhibit no abnormality of the brain at the macroscopic level^12^. WFA-positive PNNs were widely observed around GFP-positive inhibitory neurons throughout the adult brain, a finding consistent with those reported previously^3^,^13^ (Fig. 1A); however, we found it surprising that WFA-labeling and GFP fluorescence were mutually exclusive in some regions, including the dorsal tenia tecta, the piriform cortex, the lateral amygdala, the basolateral amygdala, the basomedial amygdala, the hippocampal CA2 region, the ventromedial hypothalamus, the entorhinal cortex, the temporal association cortex, and the ectorhinal cortex, even though these regions contained many GFP-positive GABAergic neurons (Fig. 1B). These PNN-expressing non-GABAergic neurons were labeled with calcium/calmodulin-dependent protein kinase II (CaMKII), a marker of excitatory neurons (Fig. 1C). Thus, PNNs are predominantly expressed around excitatory neurons rather than inhibitory neurons in these brain regions. The brain regions are associated with emotional memory formation^14^. Therefore, we reasoned that PNN-expressing excitatory neurons contribute to fear memory.

### PNN-expressing excitatory neurons are spatially clustered in the amygdala

**Figure 2:**
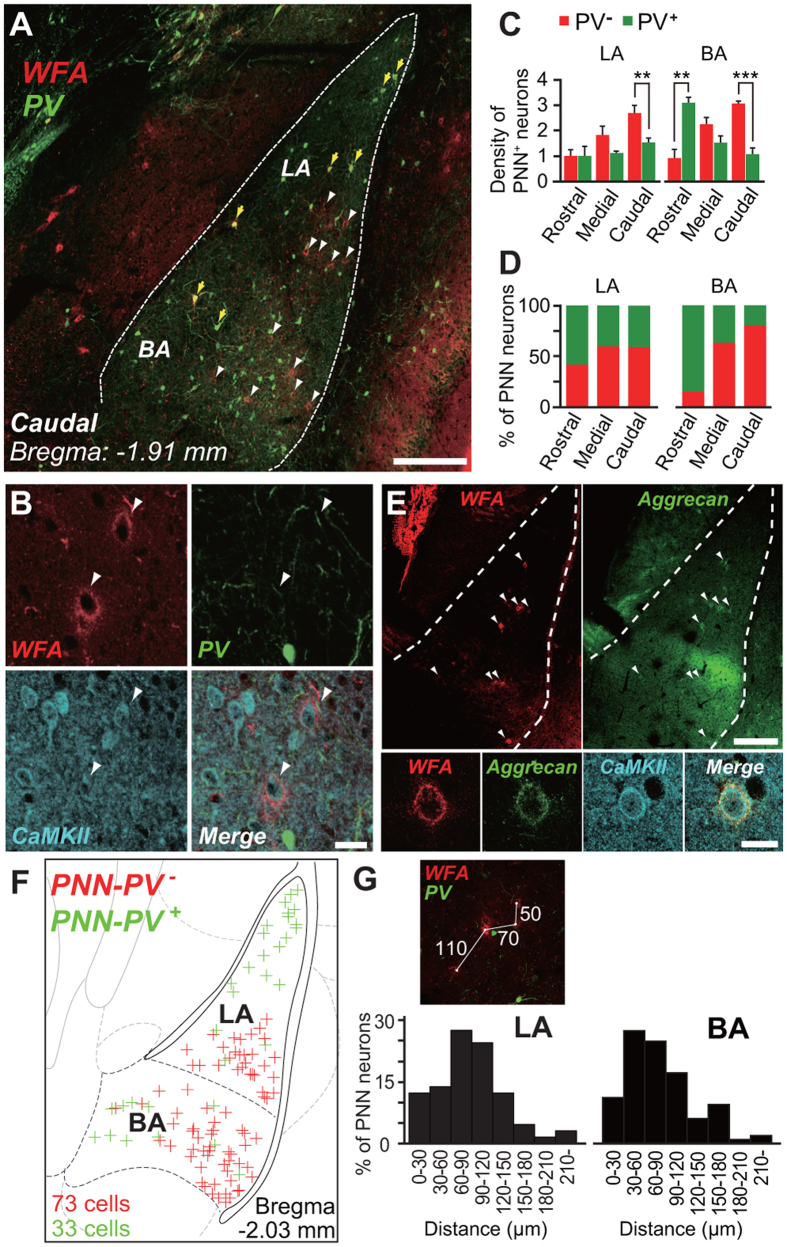
Distribution of PNNs that enwrap a subpopulation of excitatory neurons, including those positive for aggrecan immunoreactivity, in the LA and BA. (**A**) Low-magnification confocal microscopy image of cells double-labeled with WFA (red) and anti-PV antibody (green) in the caudal portions of the LA and BA. PNNs enwrap not only PV^+^ (yellow arrows) but also PV^−^ (white arrowheads) neurons. Scale bar, 200 μm. (**B**) Triple fluorescence signals in the LA generated by WFA (red), an anti-PV antibody (green), and an anti-CaMKII antibody (cyan), as well as a merged image (Merge). Arrowheads indicate that WFA is detected in neurons expressing CaMKII, but not in neurons expressing PV. *n* = 6 (91/91 neurons in 12 slices). Scale bar, 20 μm. (**C**) The average density of PNN neurons that are PV^−^ (red) or PV^+^ (green) in a single slice of one hemisphere throughout the rostrocaudal axis of the LA (left) and BA (right). Error bars indicate SEM. *n* = 9 mice, *t*-test, **P < 0.01, ***P < 0.001. (**D**) Percentage of PNN neurons throughout the rostrocaudal axis of the LA (upper) and BA (lower) that are PV^−^ (red) or PV^+^ (green). *n* = 9 mice (224 neurons in 33 slices were analyzed). (**E**) Low-magnification confocal microscopy images of double-labeling (arrowheads) for WFA (left, red) and aggrecan (right, green) in the caudal LA and BA (upper). Triple-labeled fluorescence signals generated by WFA (red), anti-aggrecan (green), and anti-CaMKII antibodies (cyan) in the LA, and a merged image (Merge) (lower). WFA/CaMKII double-labeling is detected in a neuron expressing aggrecan. *n* = 3 mice. Scale bar, 200 μm (upper) or 20 μm (lower). (**F**) Distribution of PNN-PV^−^ (red, 73 neurons) and PNN-PV^+^ (green, 33 neurons) neurons in the caudal LA and BA that were analyzed in (**D**). (**G**) Distance from a PNN-PV^−^ neuron to its nearest neighbor in the LA (left) and BA (right) constructed from 129 neurons and 209 neurons in (**F**), respectively. Upper panel, representative image analysis.

To verify the abovementioned hypothesis, we first examined the types and distribution of PNN-expressing neurons in the lateral (LA) and basolateral (BA) nuclei of the amygdala, in which plasticity occurs when sensory cues are paired with an aversive stimulus^10^. All PNN-expressing GABAergic neurons contained PV (Fig. S1A and Table S1), and all PNN-expressing PV-negative (PNN-PV^−^) neurons expressed CaMKII (Fig. 2A,B and Table S1) and neurogranin^15^ (Fig. S1B), but showed no immunoreactivity for GABA (Fig. S1C). These data demonstrate that the population of PNN neurons in the LA and BA consists of two subpopulations: PNN-PV-positive (PV^+^) inhibitory neurons and PNN-PV^−^ excitatory neurons. Therefore, an anti-PV antibody was used in the following experiments to distinguish between PNN-expressing inhibitory neurons and PNN-expressing excitatory neurons in the LA and BA. There were more PNN-PV^−^ neurons than PNN-PV^+^ neurons in these regions, except for the rostral portions of the LA and BA (Fig. 2C,D). Moreover, a large percentage of PV^+^ neurons in the medial and caudal portions of the BA were not sheathed by PNNs (Fig. S2), in contrast to previous descriptions of PV^+^ neurons in the neocortex and the hippocampus^16^,^17^. Because the proteoglycan aggrecan is a major PNN component^18^, we next examined expression of aggrecan in WFA-labeled neurons in the LA and BA. Aggrecan immunoreactivity largely overlapped with WFA-labeling (Fig. 2E, upper). High-magnification images showed that PNN-CaMKII^+^ neurons exhibited immunoreactivity for aggrecan (Fig. 2E, lower). The pattern resembled that seen for WFA (Fig. 2E, lower).

Figure 2F summarizes the distribution of PNN-PV^−^ and PNN-PV^+^ neurons in 12 brain slices obtained from nine animals. PNNs in the medial and caudal regions of the amygdala exhibited an unusual distribution pattern. Whereas a large proportion of PNN-PV^+^ neurons were localized in the dorsal LA, PNN-PV^−^ neurons were spatially concentrated in the ventral LA and in the lateral portion of the BA (Fig. 2F). The mean distance 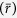 from a given PNN-PV^−^ neuron to its nearest neighbor in the LA was 89.9 ± 7.5 μm, and that in the BA was 84.9 ± 4.4 μm (Fig. 2G). Assuming that PNN-PV^−^ neurons are randomly distributed, the expected mean distance 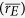 in the LA was 164.8 ± 11.4 μm and that in the BA was 169.1 ± 9.3 μm (LA: *n* = 8; *t*-test, *t*_7_ = 3.97, *P* = 0.0081; BA: *n* = 9; *t*-test, *t*_8_ = 6.04, *P* = 0.003; see Methods), suggesting that PNN-PV^−^ neurons are spatially clustered.

### PNN-expressing excitatory neurons express c-Fos during fear conditioning

**Figure 3:**
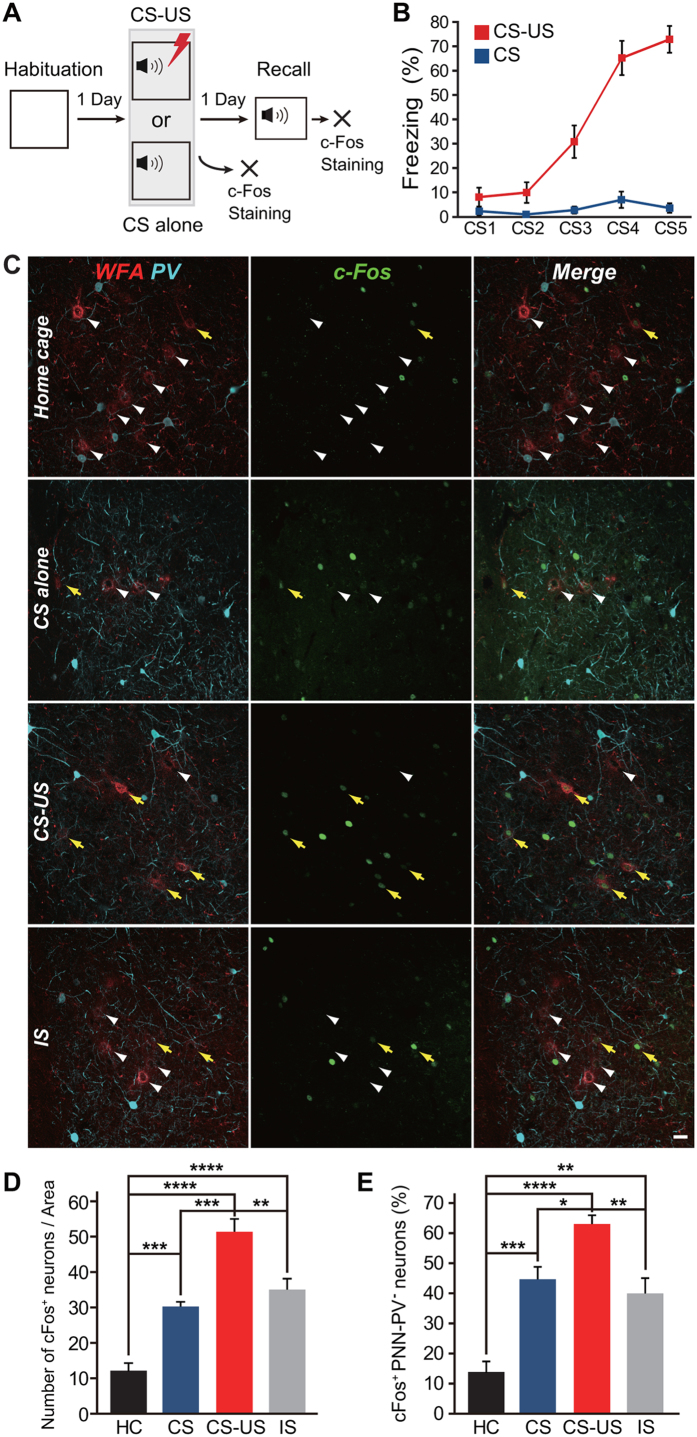
c-Fos expression in PNN-PV^−^ neurons is induced by fear learning. (**A**) The experimental schedule. (**B**) Tone-induced freezing in mice that received either tone only (blue line, *n* = 16) or tone-shock pairing (red line, *n* = 17). Error bars indicate the SEM. (**C**) Representative images showing WFA-labeling (red) and expression of PV (cyan) and c-Fos (green) in mice exposed to their home cage (HC; upper), CS-alone (middle upper), CS-US pairing (middle lower), or IS (lower). Yellow arrows indicate PNN-PV^−^ neurons expressing c-Fos. White arrowheads indicate PNN-PV^−^ neurons that do not express c-Fos. Scale bar, 20 μm. (**D**) Total number of neurons expressing c-Fos in mice subjected to HC (black, *n* = 6), CS-alone (blue, *n* = 6), paired CS-US (red, *n* = 5), and IS (gray, *n* = 6). Fear conditioning by CS-US pairing (****P < 0.0001 *vs*. HC), CS-alone training (***P < 0.001 *vs*. HC), and IS (****P < 0.0001 *vs*. HC) increases the number of neurons expressing c-Fos. (**E**) The probability of c-Fos expression in PNN-PV^−^ neurons in mice subjected to HC, CS-alone, paired CS-US, and IS. Compared with animals in their HC and those exposed to CS-alone and IS, c-Fos is localized to PNN-PV^−^ neurons in mice subjected to fear conditioning. Colors as in (**D**). Error bars indicate the SEM. *P < 0.05, **P < 0.01, ***P < 0.001, and ****P < 0.0001; one-way ANOVA with Tukey’s post hoc test.

We next measured c-Fos expression in PNN-PV^−^ neurons in the ventrolateral subdivision of the LA after fear conditioning and after a fear memory retrieval test (Fig. 3A). Mice were exposed to five pairings of a white-noise cue (conditioned stimulus, CS) either alone or combined with a mild foot shock (unconditioned stimulus, US), which resulted in an increase in freezing behavior with successive CS-US pairings, but not with CS-alone (Fig. 3B). Fear conditioning resulted in a significant increase in the total number of c-Fos^+^ neurons (51.3 ± 3.7 neurons/area) compared with that in the control groups (home cage: 12.2 ± 2.1 neurons/area, *n* = 6; one-way ANOVA, *F*_3,19_ = 32.03, *P* = 0.000000048 *vs*. CS-US; CS-alone: 30.3 ± 1.3 neurons/area, *n* = 6, *P* = 0.00026 *vs*. CS-US; immediate shock (IS): 34.4 ± 3.6 neurons/area, *n* = 6, *P* = 0.0025 *vs*. CS-US; Fig. 3C,D). The probability of c-Fos expression in the PNN-PV^−^ neurons was 63.0% ± 2.9% (home cage: 13.8% ± 3.5%, *n* = 6; one-way ANOVA, *F*_3,19_ = 19.90, *P* = 0.0000021 *vs*. CS-US; CS-alone: 44.7% ± 4.1%, *n* = 6, *P* = 0.049 *vs*. CS-US; IS: 39.7% ± 6.0%, *n* = 6, *P* = 0.0096 *vs*. CS-US; Fig. 3E). Interestingly, there was no significant difference in the probability of c-Fos expression in PNN-PV^+^ neurons between all the groups (one-way ANOVA, *F*_3,19_ = 2.66, *P* = 0.077; Fig. S3). More PNN-CaMKII^+^ neurons than CaMKII^+^ neurons without PNNs expressed c-Fos after training (WFA^+^CaMKII^+^: 61.4% ± 6.3%, *n* = 8; WFA^-^CaMKII^+^: 9.65% ± 1.2%, *n* = 8; Wilcoxon paired signed-rank test, *P* = 0.000931; Fig. S4). On the other hand, c-Fos expression did not differ between PV^+^ neurons with and without PNNs (WFA^+^PV^+^: 33.5% ± 5.4%, *n* = 6; WFA^-^PV^+^: 26.6% ± 2.0%, *n* = 6; Wilcoxon paired signed-rank test, *P* = 0.31; Fig. S4). Therefore, PNN-CaMKII^+^ neurons are more likely to be recruited during fear conditioning.

### Activation of PNN-expressing neurons correlates positively with fear memory

**Figure 4:**
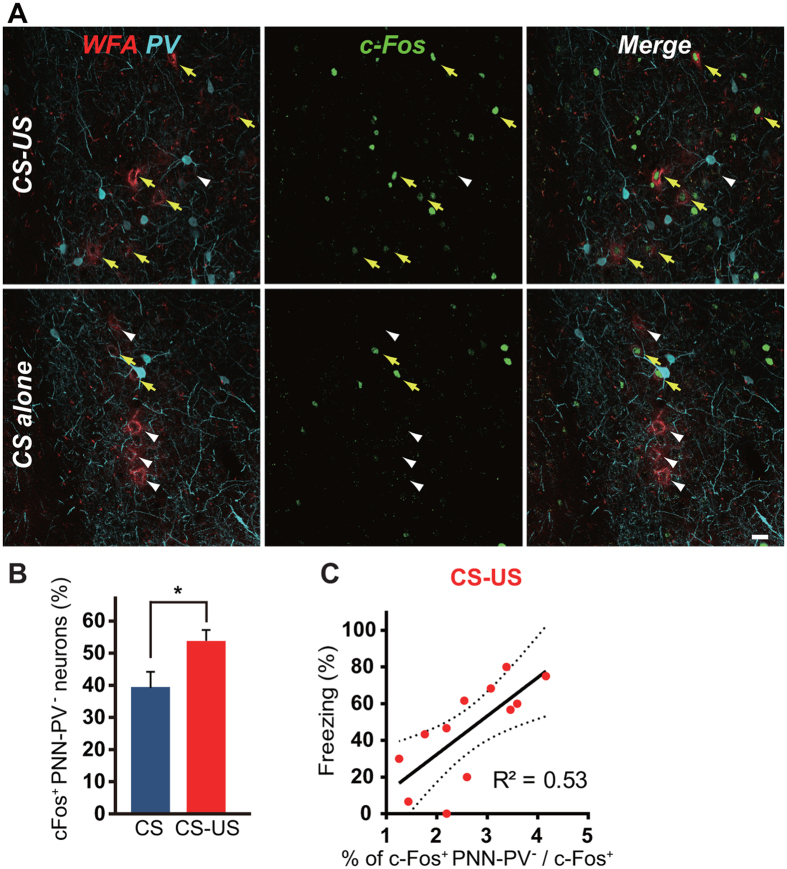
PNN-PV^−^ neurons express c-Fos during memory retrieval. (**A**) WFA-labeling (red) and expression of PV (cyan) and c-Fos (green) during the cued recall test in mice that received fear conditioning (upper) or CS-alone training (lower). Yellow arrows indicate PNN-PV^−^ neurons expressing c-Fos. White arrowheads indicate PNN-PV^−^ neurons that do not express c-Fos. Scale bar, 20 μm. (**B**) c-Fos is expressed in PNN-PV^−^ neurons during fear recall in the CS-US group (red; *n* = 12) compared with the CS-alone group (blue; *n* = 14). Error bars indicate the SEM. *W* = 44, *P < 0.05; Wilcoxon paired signed-rank test. (**C**) The normalized probability of neurons expressing PNNs correlates positively with freezing during fear recall in the CS-US group (red; *n* = 12, *P* < 0.01).

Twenty-four hours after fear conditioning, mice were re-exposed to the training cue. Fear memory recall induced c-Fos expression in PNN-PV^−^ neurons (Fig. 4A). The probability of c-Fos expression in PNN-PV^−^ neurons was higher in the CS-US group than in the CS-alone group (CS-US: 52.5% ± 3.7%, *n* = 12; CS-alone: 38.4% ± 6.0%, *n* = 14; *W* = 44, *P* = 0.042; Fig. 4B) and also in the unpaired conditioned group (paired CS-US: 41.3% ± 4.5%, *n* = 4 unpaired CS-US: 17.6% ± 4.3%, *n* = 5; *W* = 19, *P* = 0.037; Fig. S5). To exclude the possibility that expression of c-Fos in PNN-PV^−^ neurons was due to activation of neighboring neurons, the number of PNN-PV^−^ neurons expressing c-Fos was normalized to the total number of c-Fos^+^ neurons. The normalized probability of c-Fos expression in PNN-PV^−^ neurons during memory retrieval was negatively correlated with the activation of neighboring neurons in the CS-US group (Fig. S6A), but showed a weak but positive correlation in the CS-alone group (Fig. S6B). These findings indicate that PNN-PV^−^ neurons are activated after fear memory retrieval, regardless of whether surrounding neurons are activated. Importantly, the normalized probability of c-Fos expression in PNN-PV^−^ neurons correlated positively with freezing within the CS-US group (Fig. 4C), and even within both the CS-US and CS-alone groups (Fig. S6C), suggesting that greater activation of PNN-PV^−^ neurons is linked to increased fear memory.

## Discussion

To date, researchers have mainly examined the role of PNNs in inhibitory neurons^19^ since PNNs were barely detectable in association with excitatory neurons^19^,^20^,^21^,^22^. However, we found that PNNs, as determined by aggrecan expression and WFA-labeling, were present mainly in association with excitatory neurons in brain areas related to emotional memory function, suggesting that PNNs modulate the function of excitatory neurons.

PNNs limit plasticity in the adult central nervous system^2^,^23^. However, this function seems to differ depending on the brain region and animal species. For example, PNN degradation allows the induction of long-term potentiation in the hippocampal CA2 area where plasticity is normally limited^5^, and, likewise, the enhancement of long-term depression in the perirhinal cortex^8^. By contrast, the digestion of PNNs leads to the impairment of long-term potentiation at thalamo-LA synapses^7^ and the absence of enhanced plasticity in the feline visual cortex^24^. Since the types of PNN-expressing neurons differ according to the brain region, cell type-specific degradation of PNNs could provide insight into how PNNs play a role in plasticity, including fear memory.

We showed here that excitatory neurons expressing PNNs are functionally different from those lacking PNNs. PNN-PV^−^ excitatory neurons are activated during fear conditioning, although the precise mechanism remains unclear. WFA recognizes 4-O-sulfation on chondroitin sulfate chains^25^. The deposition of chondroitin 4-sulfate on neurons causes membrane depolarization, which may help neurons reach the threshold for spike firing^26^, thereby lowering the threshold of c-Fos expression. Alternatively, since the meshwork of PNNs surrounds synaptic contacts at thalamocortical boutons^25^, synaptic inputs from both the thalamus and cortex may converge on PNN-PV^−^ neurons. Our data do not rule out PNN-PV^−^ neuron activation due to sensory stimuli because exposure to tone or foot shock alone resulted in the upregulation of c-Fos in these neurons. However, PNN-PV^−^ neuron activation was affected by the activity of neighboring neurons in the CS-alone group, but not in the CS-US group. Furthermore, the population of PNN-PV^−^ neurons activated during retrieval was higher in the paired CS-US group than in the unpaired CS-US group. Hence, these neurons may encode CS-US associations. While almost nothing is known about the participation of PNN-PV^−^ neurons in memory traces, our data suggest that a memory engram is distributed in a subpopulation of neurons, PNN^+^ neurons, in the LA.

## Methods

### Animals

Male C57BL/6J mice (aged 5–10 weeks; SLC, Hamamatsu, Japan) and GAD67-GFP knock-in mice^12^ were used in this study. The mice were housed in plastic cages under a 12 h light/dark cycle at 24 °C and had free access to water and food. All procedures were approved by the animal experiment ethics committee of Hoshi University and performed in accordance with the Hoshi University guidelines for the care and use of laboratory animals. All experimental procedures minimized the number and suffering of the animals.

### Immunohistochemistry

Mice were anesthetized using intraperitoneal urethane (1.25 g/kg) and transcardially perfused with phosphate-buffered saline (PBS; pH 7.4) followed by 4% paraformaldehyde in PBS. The brains were post-fixed overnight at 4 °C in the same fixative solution, transferred to 30% sucrose in PBS for 48 h, and then cut into 30 μm coronal sections using a cryostat (CM1510-11, Leica Co., Wetzlar, Germany). The free-floating sections were blocked with 5% bovine serum albumin (BSA) and 0.3% Triton X-100 in PBS for 1 h at room temperature and incubated overnight at 4 °C with the following antibodies: mouse monoclonal anti-parvalbumin (1:5,000; P3088, Sigma-Aldrich, St. Louis, MO, USA), rabbit polyclonal anti-parvalbumin (1:3,000; PV-Rb-Af750, Frontier Institute Co., Ltd. Hokkaido, Japan), mouse monoclonal anti-CaMKIIα (1:400, Cat. no. 05-532, clone 6G9, Merck Millipore), rabbit polyclonal anti-neurogranin (1:1,000; AB5620, Merck Millipore), rabbit polyclonal anti-GABA (1:4,000; A2052, Sigma-Aldrich), rabbit polyclonal anti-aggrecan (1:1,000; AB1031, Merck Millipore), and rabbit polyclonal anti-c-Fos (1:5,000; Cat. no. 226 003, Synaptic Systems, Goettingen, Germany). The following secondary antibodies used in the study were all purchased from Thermo Fisher Scientific (Waltham, MA, USA): Alexa 488-conjugated donkey anti-mouse IgG (1:1,000; A21202), Alexa 488-conjugated donkey anti-rabbit IgG (1:1,000; A21206), Alexa 594-conjugated donkey anti-rabbit IgG (1:1,000; A21207), Alexa 647-conjugated donkey anti-mouse IgG (1:1,000; A31571), and Alexa 647-conjugated donkey anti-rabbit IgG (1:1,000; A31573). To visualize PNNs, sections were incubated overnight at 4 °C with biotin-conjugated WFA (1:3,000; BA-3101, EY laboratories, Inc., San Mateo, CA, USA), followed by Alexa 594-conjugated streptavidin (0.67 μg/ml; S11227, Thermo Fisher Scientific). Labeled sections were mounted onto microscope slides with Prolong Diamond Antifade Reagent containing DAPI (Thermo Fisher Scientific).

### Analysis of distance to the nearest neighbor

The mean distance 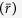
from each individual to its nearest neighbor in the LA and BA is represented as 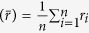, where *n* and *r*_*i*_ denote the total number of PNN-PV^−^ neurons in each region and the distance of each individual to its nearest neighbor, respectively. If this population is randomly distributed, the expected mean distance 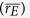
can be formulated by the following equation: where *S* is the area of the LA or BA^27^.

### Behavioral procedures

The day before fear conditioning, male C57BL/6J mice were allowed 10 min to freely explore the conditioning chamber (box A), which consisted of a transparent plastic box with a stainless steel grid floor (16 × 14 × 12 cm). For training, mice were placed in box A, and, after 2 min, they were presented with five pairings of a 30 s white-noise CS (65 dB, 1 Hz) that co-terminated with a 1 s foot shock US (0.3 mA) at a variable interval of 20–120 s. Mice in the unpaired group received explicitly unpaired training with the same number and specifications of CS and US stimuli. Thirty seconds after the final CS-US for the paired group or the final CS for the unpaired group, mice were returned to their home cage. The CS-alone group was exposed to the same protocol without the US. Mice in the IS group were given three consecutive 1 s foot shocks (0.3 mA) at an interval of 1 s immediately after placement in the conditioning chamber and quickly returned to their home cage. The next day, mice were transported to a novel chamber (box B), which consisted of a white plastic box (17 × 10 × 10 cm), and tested for freezing behavior when re-exposed to the CS. All sessions were video-recorded for automatic scoring of freezing (TimeFZ software, O’HARA & Co., LTD., Tokyo, Japan), which was defined as a period of immobility, except for respiratory-related movements, lasting for at least 2 s.

### Analysis of c-Fos

Mice were euthanized for immunohistochemical analysis 90 min after completing the behavioral task. The c-Fos-immunostained sections from −1.6 mm to −2.0 mm posterior to bregma were imaged at magnification ×20 and ×40 using an FV1200 confocal laser scanning microscope (Olympus, Tokyo, Japan). The c-Fos^+^ neurons in the ventrolateral subdivision of the LA were counted bilaterally using three slices from each mouse. The total number of c-Fos neurons in the LA was determined in an area of 59,862 ± 1,814 μm^3^ using ImageJ software (version 1.48; National Institutes of Health, Bethesda, MD, USA) and averaged within each mouse. The background fluorescence cut-off for all images was equal. The percentage of c-Fos^+^ neurons in PNN-PV^−^ neurons was calculated as the percentage of c-Fos^+^ neurons per total PNN-PV^−^ neurons in the LA, and was also normalized by the total number of c-Fos^+^ neurons in the same region.

### Statistical analysis

The results are expressed as the mean ± SEM. Statistical comparisons were analyzed with Student’s *t*-test (paired data, normal distribution), Wilcoxon paired signed-rank test (non-normal distribution), or one-way ANOVA followed by Tukey’s post hoc test (comparisons of multiple groups). Correlations were calculated using a linear correlation. No statistical methods were used to predetermine sample sizes, but the sample sizes used were similar to those generally reported in the field for similar experiments^9^,^17^. The criterion for statistical significance was set at *P* < 0.05.

## Additional Information

**How to cite this article:** Morikawa, S. *et al*. Activation of perineuronal net-expressing excitatory neurons during associative memory encoding and retrieval. *Sci. Rep.* **7**, 46024; doi: 10.1038/srep46024 (2017).

**Publisher's note:** Springer Nature remains neutral with regard to jurisdictional claims in published maps and institutional affiliations.

## Supplementary Material

Supplementary Information
