# Supplementary material for: Activation of perineuronal net-expressing excitatory neurons during associative memory encoding and retrieval
Source: Sci Rep. 2017 Apr 5;7:46024. doi: 10.1038/srep46024 (PMC5380958; doi:10.1038/srep46024)
Supplement: Supplementary Information [file srep46024-s1.pdf]

Activation of perineuronal net-expressing excitatory neurons during associative memory encoding and retrieval

Shota Morikawa,[1,2] Yuji Ikegaya,[3,4] Minoru Narita,[5] and Hideki Tamura[2*]

[1]Laboratory of Gene Regulation Research, Graduate School of Biological Sciences, Nara Institute of Science and Technology (NAIST), 8916-5, Takayama, Ikoma, Nara 630-0192, Japan

[2]Life Science Tokyo Advanced Research Center (L-StaR), Hoshi University School of Pharmacy and Pharmaceutical Sciences, 2-4-41, Ebara, Shinagawa-ku, Tokyo 142-8501, Japan

[3]Laboratory of Chemical Pharmacology, Graduate School of Pharmaceutical Sciences, University of Tokyo, 7-3-1, Hongo, Bunkyo-ku, Tokyo 113-0033, Japan

[4]Center for Information and Neural Networks, National Institute of Information and Communications Technology, Suita City, Osaka, 565-0871, Japan

[5]Department of Pharmacology, Hoshi University School of Pharmacy and Pharmaceutical Sciences, 2-4-41, Ebara, Shinagawa-ku, Tokyo 142-8501, Japan

[*]**Corresponding author**: Hideki Tamura

Tel.: +81-3-5498-5845; E-mail: h-tamura@hoshi.ac.jp

Table S1: Overlap of markers in PNN-PV$^-$ and PNN-PV$^+$ neurons in the LA.

	Marker/PNN-PV$^-$	Marker/PNN-PV$^+$
**GAD67-GFP**	**0/92 cells (0%)**	**80/80 cells (100%)**
**CaMKII**	**121/121 cells (100%)**	**0/30 cells (0%)**

Quantification analysis of PNN-PV$^-$ and PNN-PV$^+$ neurons in the LA that express GFP in GAD67-GFP knock-in mice or CaMKII in C57BL/6J mice.

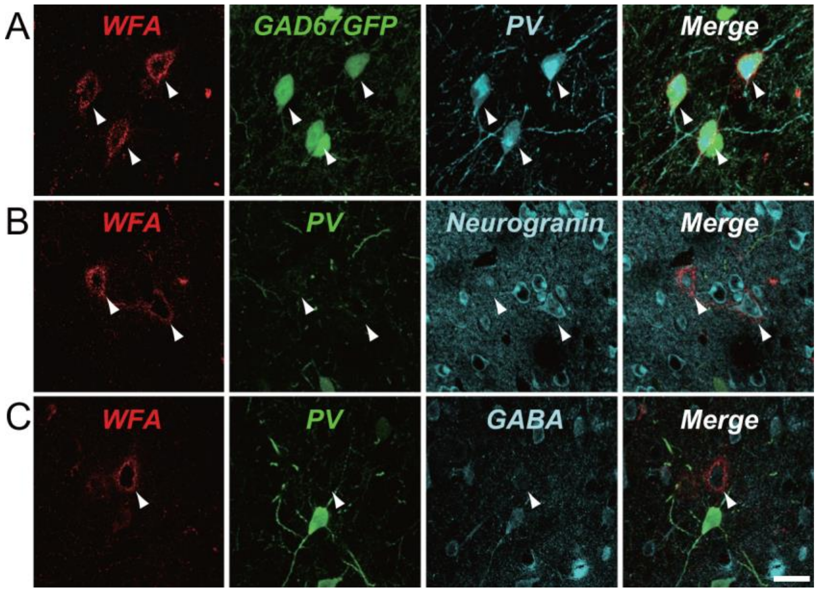

Figure S1: PNN-PV$^-$ neurons are excitatory neurons. (A) Triple-labeled fluorescence images in the LA showing signals from WFA (red), GFP (green), and an anti-PV antibody (cyan), and their merged images (Merge). Arrowheads indicate that WFA/GFP double-labeling is observed in neurons expressing PV. (B, C) Triple-labeled fluorescence images showing signals in the LA from WFA (red) and anti-PV (green) and anti-neurogranin (cyan; B) or anti-GABA (cyan; C) antibodies, and their merged images in each row (Merge). Arrowheads indicate that neurogranin, but not GABA, is expressed in PNN-PV$^-$ neurons. Scale bar, 20 μm.

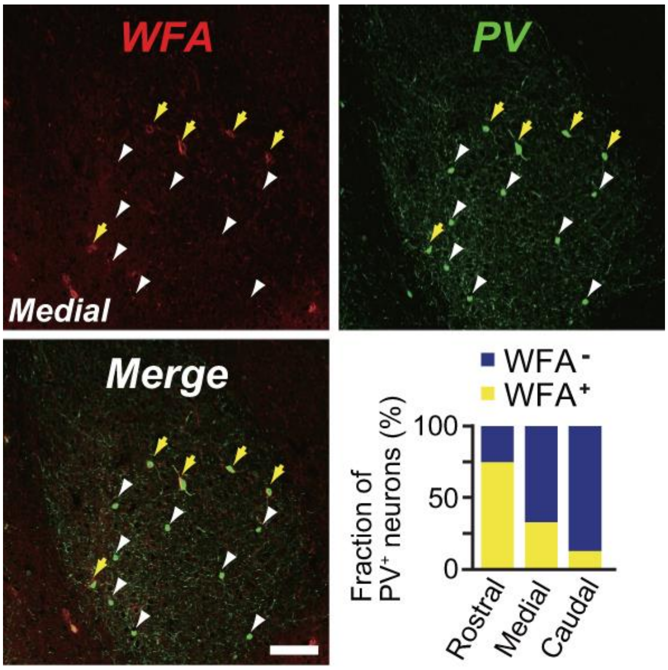

Figure S2: Representative images showing signals from WFA (red) and an anti-PV antibody (green), and their merged images (Merge), in the medial portion of the BA. Yellow arrows and white arrowheads indicate PV^+ neurons that are enwrapped or not enwrapped by PNNs, respectively. Scale bar, 100 μm. The graph shows the percentage of PV^+ neurons in each BA region that is enwrapped (yellow) or not enwrapped (blue) by PNNs. A large population of PV^+ neurons in the medial and caudal portions of the BA is not surrounded by WFA-labeled PNNs. n = 3–6 mice (804 neurons were analyzed).

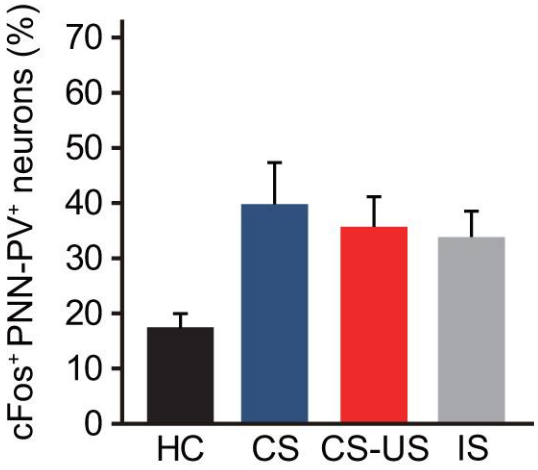

Figure S3: The probability of c-Fos expression in PNN-PV$^+$ neurons in mice subjected to HC (black; *n* = 6), CS-alone (blue; *n* = 6), paired CS-US (red; *n* = 5), and IS (gray; *n* = 6). There was no significant difference between all the groups (one-way ANOVA, $F_{3,19}$ = 2.66, *P* = 0.077). Error bars indicate the SEM.

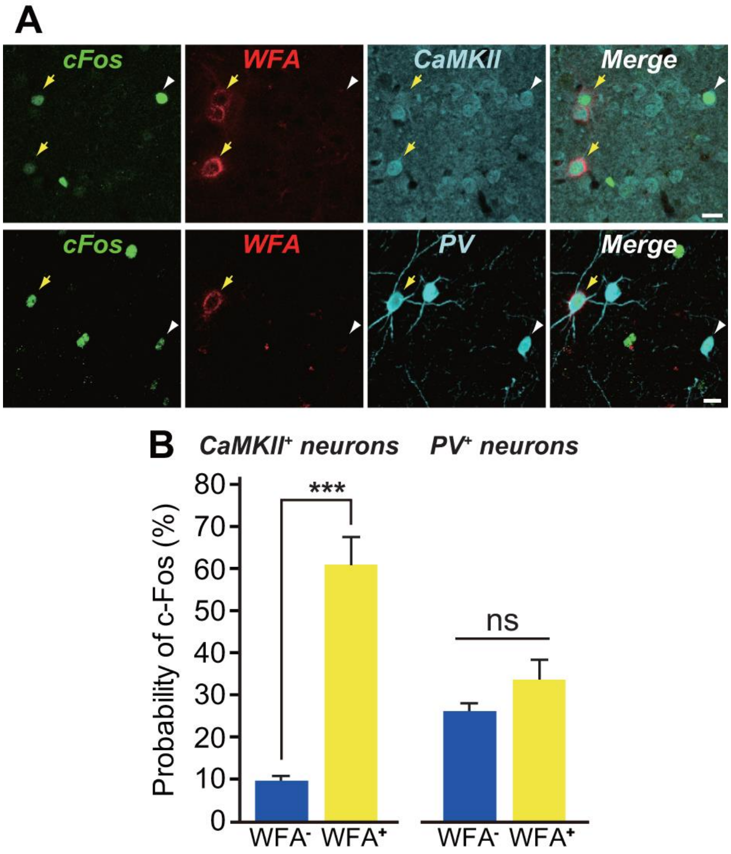

Figure S4: PNN-CaMKII$^+$ neurons are more likely than their neighbors to express c-Fos during fear conditioning. (A) Representative images showing signals from an anti-c-Fos antibody (green), WFA (red), and an anti-CaMKII (cyan, upper) or anti-PV (cyan, lower) antibody, and their merged images (Merge). Yellow arrows indicate WFA$^+$CaMKII$^+$ or WFA$^+$PV$^+$ neurons expressing c-Fos. White arrowheads indicate WFA$^-$CaMKII$^+$ or WFA$^-$PV$^+$ neurons expressing c-Fos. Scale bar, 20 μm. (B) c-Fos expression is more likely in PNN-CaMKII$^+$ neurons (left, yellow) than in neighbors lacking PNNs (blue), but is not seen in PNN-PV$^+$ neurons (right). Error bars indicate the SEM. $n = 8$, $W = 64$, $^{***}P < 0.001$; Wilcoxon paired signed-rank test.

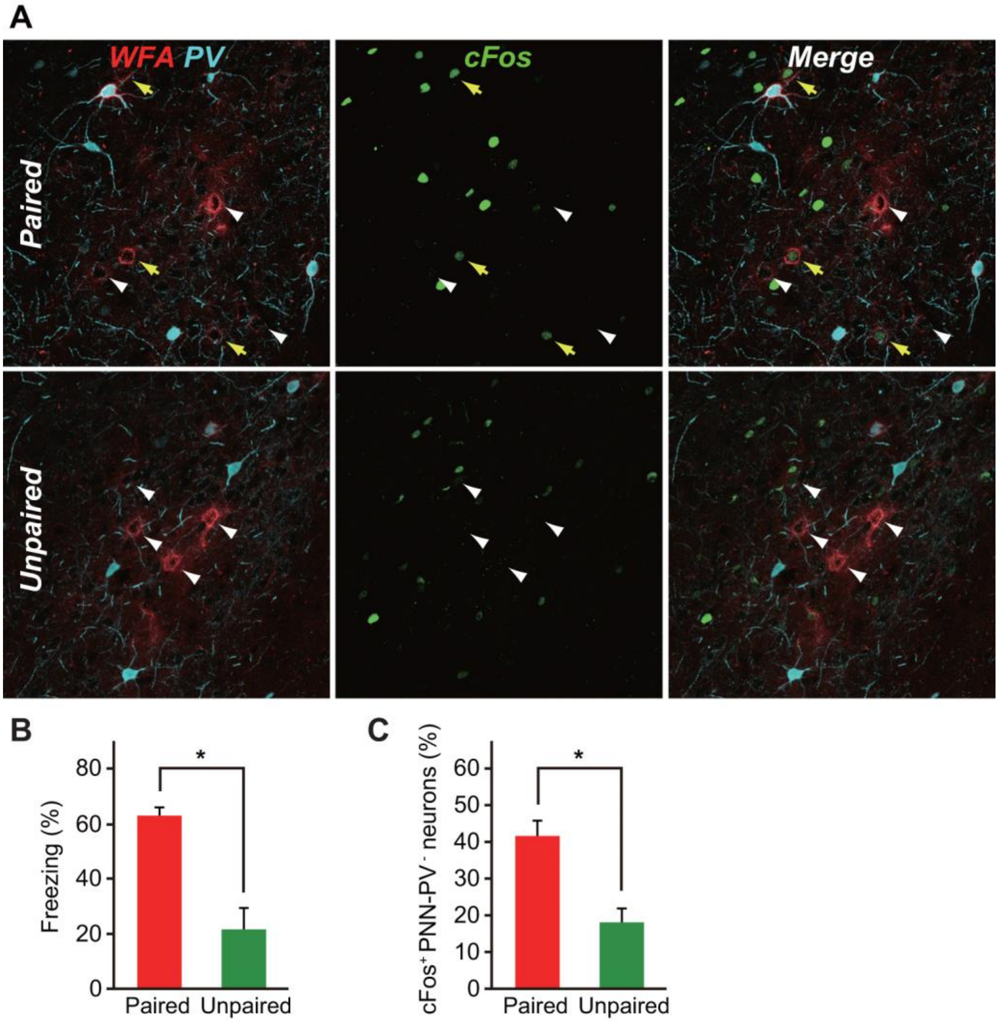

Figure S5: (A) WFA-labeling (red) and expression of PV (cyan) and c-Fos (green) during the cued recall test in mice that received paired CS-US (upper) or unpaired CS-US training (lower). Yellow arrows indicate PNN-PV$^-$ neurons expressing c-Fos. White arrowheads indicate PNN-PV$^-$ neurons that do not express c-Fos. Scale bar, 20 μm. (B) Tone-induced freezing during fear recall in mice that received either paired CS-US (red; n = 4) or unpaired CS-US (green; n = 5). (C) c-Fos is expressed in PNN-PV$^-$ neurons during fear recall in the paired group (red; n = 4) compared with the unpaired group (green; n = 5). Error bars indicate the SEM. $^*P < 0.05$; Wilcoxon paired signed-rank test.

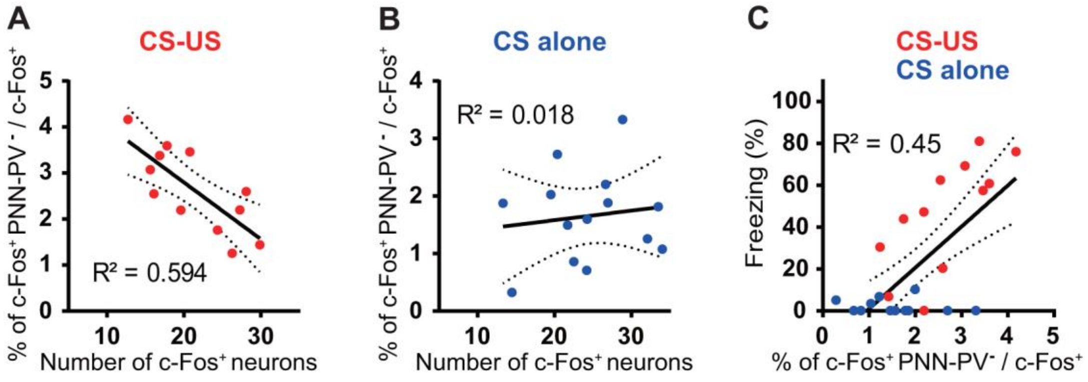

Figure S6: The normalized probability of neurons expressing PNNs is significantly correlated with the number of c-Fos$^+$ neurons during fear recall in the CS-US group (A; red; n = 12, P < 0.004), but not during CS exposure in the CS-alone group (B; blue; n = 14, P > 0.6). (C) The normalized probability of neurons expressing PNNs correlates positively with freezing during fear recall within both the CS-US (red; n = 12) and CS-alone (blue; n = 14) (P = 0.0002) groups.
